# Testing the Associations between Coping, Mental Health, and Satisfaction with Life in Portuguese Workers

**DOI:** 10.3390/ejihpe13070092

**Published:** 2023-07-11

**Authors:** Filipe Rodrigues, Pedro Morouço, Tiago Santos

**Affiliations:** 1 ESECS—Polytechnic of Leiria, 2411-901 Leiria, Portugal; pedro.morouco@ipleiria.pt; 2 Life Quality Research Centre (CIEQV), 2040-413 Leiria, Portugal; 3 Center for Innovative Care and Health Technology (CiTechcare), 2410-541 Leiria, Portugal; 4 National School of Public Health (ENSP—UNOVA), NOVA University Lisbon, 1600-560 Lisbon, Portugal; tiagosantos@esdrm.ipsantarem.pt

**Keywords:** cope, mental health, workers, satisfaction with life, Portugal

## Abstract

The present study aimed to explore the relationships between coping strategies, symptoms of depression, anxiety, and stress, and satisfaction with life among Portuguese workers. A sample of 402 participants (207 male, 195 female), ranging in age from 18 to 70 years (M = 32.90, SD = 11.75), was included in the study. Participants reported varying levels of work experience, ranging from 1 to 45 years (M = 10.62, SD = 4.07). The sample encompassed diverse occupations, including arts and design (n = 28), engineering (n = 23), marketing (n = 27), administration (n = 50), transportation and logistics (n = 57), clerks (n = 63), lawyers (n = 21), factory workers (n = 20), accountant and finance (n = 41), journalism (n = 27), health care (n = 29), and others (n = 16). To examine the associations between each determinant and satisfaction with life, hierarchical multiple regression analyses were conducted. Two models were tested, with predictors entered in blocks based on theoretical and empirical considerations. The second model accounted for 52.4% of the variance in satisfaction with life (F (14, 384) = 3.884, *p* < 0.001, *R*^2^ = 0.27, adjusted *R*^2^ = 0.24). Depressive symptoms and stress consistently exhibited a significant association (*p* < 0.05) with satisfaction with life across all tested models. In terms of coping mechanisms, instrumental support reinterpretation, disengagement, and humor demonstrated a significant association with satisfaction with life (*p* < 0.05). The findings suggest that employing adaptive coping strategies may help mitigate symptoms of mental distress and enhance satisfaction with life. By understanding the relationships between coping strategies, mental health symptoms, and satisfaction with life, interventions can be developed to promote well-being and improve overall quality of life among Portuguese workers.

## 1. Introduction

Psychosocial responses to pandemic outbreaks have been recognized as potential triggers for both immediate and long-term mental health consequences [1]. The experience of mental distress in the context of such crises can be attributed to various factors, including workplace challenges, which may contribute to failures and inaccuracies, and pose a significant risk for severe psychological issues detrimental to individuals’ well-being and overall health, such as severe depression, mental burnout, and even thoughts of suicide [2,3]. To mitigate the impact of these stressors, individuals employ coping mechanisms to manage the demands imposed by stressful events that are perceived as overwhelming or surpassing one’s available resources [4]. It is important to note that not all coping strategies are equally effective, as some may exacerbate psychological distress, while others can effectively attenuate negative psychological responses to stressors, particularly during circumstances such as lockdowns. Recognizing contentment as a key indicator of mental well-being and deriving meaning in life is paramount. Positive well-being can significantly influence individuals’ thoughts and emotions in adverse situations, driving their resilience and determination to achieve desired goals and ultimately leading to an enhanced quality of life. Enhancing our understanding of the benefits of different coping strategies is crucial, as it can empower individuals to effectively manage negative psychological responses to stressful events. Consequently, establishing evidence of the association between coping mechanisms and mental distress can provide valuable insights for researchers developing mental health interventions aimed at fostering satisfaction with life among workers [5].

### 1.1. Psychological Distress

Extensive research has demonstrated a notable increase in stress, depression, and anxiety levels during the pandemic [1,5,6]. Moreover, the population experienced a higher prevalence of mental health issues during the lockdown period compared to the pre-pandemic period [7,8,9]. Psychological or mental distress encompasses various dimensions that impact an individual’s functioning, with symptoms such as stress, anxiety, and depression being manifestations of this distress [10]. Depressive symptoms are characterized by a diminished sense of self-esteem and motivation, accompanied by a perceived low likelihood of achieving personally valuable aspirations [11]. Anxiety symptoms, on the other hand, involve a persistent fear of anticipated events and the apprehension of failing to meet expectations [12]. Stress symptoms encompass enduring states of agitation, tension, low frustration tolerance, and distorted thinking patterns [13].

Extensive research has consistently demonstrated the negative association between experienced psychological distress and well-being, as well as quality of life, across various populations during and post-pandemic, including students [14], individuals engaging in physical exercise [6], the general population [15], university workers [16], and hospitalized patients [17]. The strategies individuals employ to adapt and navigate adverse circumstances and psychological distress have the potential to influence positive mental health and well-being. These coping mechanisms can either exacerbate or alleviate mental distress, shaping the trajectory toward positive mental health outcomes [18]. There is evidence indicating that coping strategies are associated with symptoms of depression, anxiety, and stress, with the efficacy of these strategies varying among individuals [19]. Therefore, shedding light on coping factors that can mitigate mental health distress in the workplace is of utmost importance, as it can empower workers to cultivate the mental well-being necessary for optimal performance and satisfaction in their professional endeavors [5]. Recognizing and engaging in adaptive coping strategies, characterized by persistence and effort in pursuing desired goals and effectively navigating undesired or traumatic events, holds substantial potential to significantly enhance overall quality of life.

### 1.2. Coping Mechanisms as Resources for Managing Mental Health Distress

Coping mechanisms, defined as purposeful actions taken to manage the demands of stressful events [20], have been widely acknowledged for their significant impact on stress-related mental and physical health outcomes, as well as their potential for interventions aimed at increasing well-being and improving quality of life [5]. Coping involves conscious efforts to prevent or reduce threats, damage, loss, or associated psychological distress [19]. The authors highlight the complexity of identifying the nature of coping reactions, as they may initially be strategic and intentional but can become automatic with repetition. Consequently, individuals facing adverse events actively employ various coping strategies to alleviate the source of stress [19].

Numerous authors [4,21] have proposed the existence of 14 coping mechanisms, namely humor, positive reframing, emotional social support, acceptance, religion, instrumental support, planning, active coping, behavioral disengagement, self-blaming, substance use, venting, self-distraction, and denial. Carver et al. [20] categorize humor, positive reframing, emotional social support, acceptance, and religion as emotion-focused coping strategies, while instrumental support, planning, and active coping are classified as problem-focused strategies. In contrast, behavioral disengagement, self-blaming, substance use, venting, self-distraction, and denial are considered dysfunctional coping strategies [20]. Substantial evidence also supports the distinction between adaptive and maladaptive coping strategies. Adaptive coping, as proposed by several authors [18,22,23], encompasses acceptance, active coping, positive reframing, planning, religion, seeking social support, emotional and instrumental support, and humor. Conversely, maladaptive coping includes behavioral disengagement, venting, denial, substance use, self-distraction, and self-blame.

Existing research [5,18,24] provides evidence for positive relationships between adaptive coping mechanisms and favorable outcomes such as well-being and life satisfaction. Conversely, maladaptive coping strategies have been found to correlate with negative outcomes such as depression, anxiety, stress, and negative affect. Moreover, problem-oriented coping has shown positive associations with adaptive outcomes, while emotion-oriented coping, largely dysfunctional in nature, has exhibited negative associations with adaptive outcomes [20].

### 1.3. Literature Review and Hypotheses Development

Despite the imperative to comprehend how workers cope with mental distress resulting from past lockdowns, the investigation of coping within this population and its associations with mental health and well-being remains limited [5]. While some research has explored the relationship between coping strategies and mental health indicators [16,25,26], there is a paucity of information regarding the hypothesis that adaptive coping strategies function as a mechanism to mitigate psychological distress indicators, consequently fostering greater life satisfaction, especially among workers who have experienced significant negative effects on mental health due to social isolation [6]. Moreover, to the best of our knowledge, existing studies have predominantly examined general dimensions of coping [25], thereby failing to elucidate the individual contributions of each coping mechanism in ameliorating symptoms of depression, anxiety, and stress, and their associations with life satisfaction.

The present study aims to investigate the associations between coping mechanisms, mental health outcomes, and satisfaction with life among Portuguese workers. Notably, we explore the connections between coping, depressive symptoms, anxiety, stress, and an indicator of well-being within an understudied group—workers—who place considerable importance on life satisfaction due to the challenges they have faced during the pandemic and their ongoing quest for mental recovery and well-being. Despite the daily challenges associated with their work conditions, these individuals have endured prolonged periods of downtime while maintaining the necessary commitment to perform effectively. Many workers likely employ high levels of adaptive coping strategies, as their future prospects for a better quality of life are instrumental in attaining their goals and meeting their expectations. By examining the relationship between each coping strategy, psychological distress, and life satisfaction, this study will contribute to the existing literature in health psychology [5,27,28]. Previous studies conducted among workers have typically treated coping strategies as a unifying construct rather than considering them as multiple dimensions of mental health [25]. Based on this rationale, we propose the following hypotheses:

**Hypothesis 1** **(H1).** *Symptoms of depression, anxiety, and stress would be negatively associated with life satisfaction.*

**Hypothesis 2** **(H2).** *Adaptive coping strategies would be negatively associated with symptoms of depression, anxiety, and stress.*

**Hypothesis 3** **(H3).** *Maladaptive coping strategies would be positively associated with symptoms of depression, anxiety, and stress.*

**Hypothesis 4** **(H4).** *Adaptive coping, but not maladaptive coping, would be positively associated with life satisfaction.*

## 2. Materials and Methods

### 2.1. Participants

The a priori sampling calculator for hierarchical multiple regression analysis [29] was used to calculate the minimum sample size required for this study to be valid and reliable. The following inputs were used: anticipated effect size for set B = 0.15 (medium effect); desired statistical power = 0.95; number of predictors in set A = 3; number of predictors in set B = 14; probability level = 0.05. The results suggest that the minimum number of participants should be 198 for the results to be valid and reliable.

Data were collected between July and December of 2021. The sample consisted of 402 participants (207 male, 195 female) aged 18–70 years old (M_age_ = 32.90, SD = 11.75). Participants all reported working experience ranging from 1 to 45 years (M_exp_ = 10.62, SD = 4.07). They represented a variety of labors such as arts and design (n = 28), engineering (n = 23), marketing (n = 27), administration (n = 50), transportation and logistics (n = 57), clerks (n = 63), lawyers (n = 21), factory workers (n = 20), accountant and finance (n = 41), journalism (n = 27), health care (n = 29), others (n = 16). For inclusion, we considered those who met the following inclusion criteria: (i) Aged 18 years or older; (ii) Active employee or employer with at least 1 year of working experience, since these individuals had been working during the lockdown period; (iii) Provide informed consent to participate.

### 2.2. Instruments

Coping mechanisms in the work context were measured using the Brief Cope Portuguese version [30]. This version contains 28 items distributed across 14 factors. Each coping strategy is measured using two items (humor, item example: *I have been making jokes about it*; positive reframing, item example: *I have been trying to see it in a different light, to make it seem more positive*; emotional social support, item example: *I have been getting emotional support from others*; acceptance, item example: *I have been accepting the reality of the fact that it has happened*; religion, item example: *I have been trying to find comfort in my religion or spiritual beliefs*; instrumental support, item example: *I have been getting help and advice from other people*; planning, item example: *I have been trying to come up with a strategy about what to do*; active coping, item example: *I have been concentrating my efforts on doing something about the situation I am in*; behavioral disengagement, item example: *I have been giving up trying to deal with it*; self-blaming, item example: *I have been criticizing myself*; substance use, item example: *I have been using alcohol or other drugs to make myself feel better*; venting, item example: *I have been saying things to let my unpleasant feelings escape*; self-distraction, item example: *I have been turning to work or other activities to take my mind off things*; and denial, item example: *I have been refusing to believe that it has happened*). Participants answered each item with a Likert-type response scale ranging from 1 (I never do that) to 5 (I always do that). Mean scores were calculated for each coping factor. The results from the confirmatory factor analysis in this study support the measurement model with 14 correlated factors (Comparative Fit Index = 0.951, Tucker–Lewis Index = 0.939, Standard Mean Root Square Residual = 0.051, Root Mean Square Error of Approximation = 0.057 (90% confidence interval = [0.040, 0.064]).

The Depression Anxiety Stress Scale Portuguese version [31] was used to measure symptoms of depression (item example: *I couldn’t seem to experience any positive feeling at all*), anxiety (item example: *I was aware of dryness of my mouth*), and stress (item example: I found it hard to wind down). This scale contains 21 items (7 items per factor), and each item is scored from 0 (did not apply to me at all) to 3 (applied to me very much or most of the time). Mean scores were calculated for each factor. Results from the confirmatory factor analysis in this study support the measurement model showing acceptable scores of validity (Comparative Fit Index = 0.917, Tucker–Lewis Index = 0.910, Standard Mean Root Square Residual = 0.054, Root Mean Square Error of Approximation = 0.073 (90% confidence interval = [0.067, 0.088]).

The Satisfaction with Life Scale Portuguese version [18] was used to measure perceived satisfaction with life. This scale contains 5 items (item example: *In most ways my life is close to my ideal*), and each item is scored using a Likert-type scale anchored from 1 (Strongly disagree) to 5 (Strongly agree). Results from confirmatory factor analysis in this study support the measurement model showing acceptable scores of validity (Comparative Fit Index = 0.901, Tucker–Lewis Index = 0.897, Standard Mean Root Square Residual = 0.052, Root Mean Square Error of Approximation = 0.047 (90% confidence interval = [0.038, 0.068]).

### 2.3. Procedures

Ethical institutional approval (CE/IPLEIRIA/17/2021) was obtained prior to conducting this study. This study is part of the Cope@Work Project, approved by the Research Committee of the Life Quality Research Center (UID/CED/04748/2020). All procedures were performed in accordance with the ethical standards of the institutional and national research committee and with the 1964 Helsinki declaration and its later amendments or comparable ethical standards. Following ethical institutional approval, we utilized convenience sampling as a means of data collection by targeting companies within the relevant industry. We personally visited the selected companies and directly contacted the managers or relevant personnel, explaining the purpose and significance of our study. We sought their approval and cooperation, and upon their willingness to participate, we proceeded to distribute an online questionnaire to all employees within those companies.

The decision to employ convenience sampling was based on practical considerations and the accessibility of the target population, as it allowed us to gather data efficiently. However, it is important to acknowledge that this sampling approach may impose limitations on the interpretation of our findings. The companies included in the study were selected based on their availability and willingness to participate, which means the sample may not entirely reflect the entire population of interest. Therefore, caution should be exercised when interpreting the results, as they may not be fully representative of the broader industry or population.

Objectives and data collection procedures were explained individually to the managers, and, after approval, potential participants were contacted using an internal mailing list. They were invited to participate voluntarily in this study, and signed informed consent was obtained individually. Participants completed measures using a self-administered online questionnaire, which ensured anonymity and confidentiality of their responses to encourage honest and unbiased answers. The mean time to complete the questionnaires was less than 15 min.

### 2.4. Statistical Analysis

All analyses were conducted in IBM SPSS Statistics Version 26.0. (IBM Corp., Armonk, NY, USA). We used the expectation-maximization approach to handle missing completely at random data. Descriptive statistics were reported and, to determine possible deviation from normal distribution, the skewness and kurtosis estimates were divided by their corresponding standard error to get the z-score. Z-score below |1.96| suggest normal distribution [32]. Bivariate correlations were conducted considering variables of interest. Partial correlations were also performed controlling for sex. The significance level was set at *p* ≤ 0.05 to reject the null hypothesis. Alpha coefficients for internal consistency were calculated considering as acceptable coefficients ≥ 0.70 [33].

Hierarchical multiple regression analyses were performed to test the proposed relationships. Before performing the regression analysis, the tolerance test and variance inflation factor (VIF) values were analyzed to test for possible multicollinearity problems [32]. The tolerance test of the independent variables should be greater than 0.1 and VIF scores should be less than 5 to avoid multicollinearity. The Durbin–Watson statistic test for autocorrelation was also calculated, assuming an acceptable range of 1.50–2.50 [34]. We used the stepwise procedure as we intended to add variables following theoretical assumptions. Satisfaction with life was inserted in the model as the dependent variable. In Model 1, depressive, anxiety, and stress symptoms were inserted in the model, and, in Model 2, we additionally inserted in the model all coping mechanisms. Models were compared using the *R*^2^ and changes in the *R*^2^ were analyzed using the significance level at *p* ≤ 0.05 to reject the null hypothesis.

## 3. Results

### 3.1. Preliminary Results

Descriptive statistics, bivariate correlations, and internal consistency coefficients are shown in Table 1. Mean scores for planning were greater than all other coping mechanisms. Self-blame showed the greatest mean related to maladaptive coping compared to other strategies. Skewness and kurtosis values were below the cut-off indicating a normal distribution. Several significant bivariate correlations emerged as expected, namely: (a) Adaptive coping and problem-focused coping mechanisms were positively correlated (*p* < 0.01) with satisfaction with life; (b) Maladaptive coping and dysfunctional coping mechanisms were negatively correlated (*p* < 0.01) with satisfaction with life; (c) Measures of general mental distress were negatively and significantly correlated (*p* < 0.01) with satisfaction with life. Partial correlations displayed similar results compared to those from the bivariate correlations, since significance levels were maintained in all tested associations. Alpha coefficients were close to or above 0.70, showing acceptable internal consistency.

### 3.2. Hierarchical Multiple Regression Analysis

The result of the hierarchical multiple regression analysis is shown in Table 2. The tolerance values ranged from 0.32 to 0.85 and the VIF values ranged from 1.18 to 3.17. Thus, there were no multicollinearity issues in this analysis. The Durbin–Watson test yielded a value of 1.51, indicating an acceptable value close to zero for autocorrelation. We tested the significance (*p*-value) of the model to examine whether the model was significantly different from a null hypothesis. We checked the *R*^2^ value to see how much of the variance in satisfaction with life was explained by the model. To determine which variable contributed most to the model, we examined the standardized coefficients and the significance of the independent variables. In hierarchical multiple regression analysis, we compared the models when variables were added (changes in *R*^2^).

In Model 1, the regression model was statistically significant and showed that depressive symptoms, anxiety, and stress symptoms accounted for 41.4% of the variance in satisfaction with life (F [3, 398] = 27.46, *p* < 0.001, *R*^2^ = 0.17, adjusted *R*^2^ = 0.16). Model 2 accounted for 52.4% of variance in satisfaction with life (F [14, 384] = 3.884, *p* < 0.001, *R*^2^ = 0.27, adjusted *R*^2^ = 0.24). The change in *R*^2^ between the first and second model (Δ*R*^2^ = 0.10) was statistically significant (*p* < 0.05). These results indicate that the second model is the more parsimonious compared to the previous model. Depressive symptoms and stress maintained a significant association (*p* < 0.05) with satisfaction with life in both models. Regarding the significant contribution of coping mechanisms, instrumental support, reinterpretation, disengagement, and humor displayed a significant association with satisfaction with life (*p* < 0.05).

## 4. Discussion

The aim of this study was to test the associations between coping, mental health, and satisfaction with life in Portuguese workers. Based on current results, mean scores for adaptive coping and problem-focused coping were greater compared to maladaptive coping strategies, emotion-focused, and dysfunctional coping, respectively. Several significant bivariate correlations emerged, namely the significant association between adaptive coping and problem-focused coping mechanisms and satisfaction with life. In addition, maladaptive coping and dysfunctional coping mechanisms were negatively correlated with satisfaction with life. Hierarchical multiple regression analysis suggests that psychological distress indicators and disengagement have a significant negative contribution on satisfaction with life. However, instrumental support, reinterpretation, and humor may be crucial for this population to feel a higher perception of achieved goals, and self-concept, in which workers positively evaluate the overall quality of life.

In line with H1, the results revealed a negative and significant correlation between symptoms of depression, anxiety, and stress and life satisfaction, consistent with findings by Almeida et al. [5]. This suggests that increased levels of depressive symptoms are associated with a decreased perception of satisfaction with life. Santos et al. [25] also found similar results, indicating that workers with higher levels of depressive symptoms and stress exhibit lower levels of optimism regarding the future, which is an indicator of well-being. The association between symptoms of depression, anxiety, and stress with life satisfaction can be understood through theoretical frameworks that emphasize the impact of mental distress on subjective well-being. According to cognitive theories of well-being, individuals experiencing higher levels of depressive symptoms, anxiety, and stress may exhibit cognitive biases and negative thought patterns that influence their perception of life satisfaction. These cognitive biases, such as negative rumination and distorted thinking, can overshadow positive aspects of life and contribute to a diminished sense of well-being. Therefore, it can be inferred that individuals who tend to overreact to situations, experience heightened nervous energy, and struggle to relax may have lower levels of well-being, particularly satisfaction with life.

The negative association between adaptive and problem-focused coping strategies and symptoms of depression and stress can be explained by theoretical perspectives that emphasize the role of effective coping mechanisms in buffering the impact of stressors on mental health. Adaptive coping strategies, such as seeking social support, problem-solving, and maintaining a positive outlook, are theorized to facilitate effective stress management and psychological well-being. Individuals who actively engage in adaptive coping are more likely to experience a sense of control, optimism, and self-efficacy, which can mitigate the development of depressive symptoms and stress. This theoretical framework is supported by previous empirical research [26,27] and is consistent with our findings. Our study adds to the existing evidence by highlighting the importance of adaptive coping in promoting mental health and well-being among workers, underscoring the significance of employing active and constructive strategies to navigate stressors effectively.

The positive association between maladaptive coping strategies, such as emotion-focused coping and dysfunctional coping, and symptoms of depression, anxiety, and stress aligns with theoretical perspectives that emphasize the detrimental effects of ineffective coping on mental health outcomes. Maladaptive coping strategies, characterized by avoidance, denial, and ineffective emotion regulation, are theorized to impede individuals’ ability to effectively cope with stressors, leading to heightened emotional distress. Theoretical frameworks propose that these maladaptive coping mechanisms can perpetuate negative emotional states and contribute to the development or exacerbation of symptoms of depression, anxiety, and stress. Our findings are consistent with previous studies [5,14,16], further supporting the notion that individuals who struggle to cope adaptively and fail to structure their response to stressors may be more prone to experiencing psychological distress. These results underscore the importance of addressing maladaptive coping patterns and promoting more effective coping strategies to enhance workers’ mental well-being.

The positive association between specific adaptive coping strategy subscales (instrumental support, reinterpretation, and humor) and satisfaction with life can be explained by theoretical frameworks that emphasize the role of these coping mechanisms in promoting positive affect, resilience, and overall well-being. Instrumental support, which involves seeking assistance and guidance from others, can provide individuals with a sense of security, validation, and problem-solving resources, ultimately contributing to greater life satisfaction. Reinterpretation refers to the ability to find positive meaning or alternative perspectives in challenging situations, allowing individuals to reframe their experiences and cultivate a more positive outlook. Humor, as an adaptive coping strategy, can provide emotional relief, enhance social connections, and foster resilience in the face of adversity. Theoretical frameworks suggest that these adaptive coping mechanisms can promote positive affect, increase psychological flexibility, and contribute to overall life satisfaction. Our findings support the existing literature [5,16], highlighting the importance of these adaptive coping factors in facilitating greater satisfaction with life. By examining the individual contributions of each coping subscale, our study provides a nuanced understanding of the specific coping strategies that can enhance workers’ well-being.

The theoretical foundations and empirical evidence discussed for each hypothesis (H1–H4) shed light on the mechanisms underlying the observed associations. The discussion highlights the role of mental distress, coping strategies, and life satisfaction in the context of workers’ well-being, emphasizing the importance of addressing maladaptive coping patterns and promoting adaptive coping strategies to enhance psychological well-being and overall life satisfaction.

### Strengths, Limitations, and Agenda for Future Research

This study represents a significant contribution as the first examination of coping strategies, psychological distress, and satisfaction with life in a sample of Portuguese workers. A notable strength of our study is the inclusion of participants from various labor activities, allowing for a heterogeneous representation and similar perceptions regarding the variables under analysis. Furthermore, validated measures specifically designed for the adult Portuguese working population were employed, enhancing the robustness of our findings. However, it is important to acknowledge the limitations of our study. Firstly, data had to be collected from participants across different labor activities to achieve sufficient statistical power for the proposed analyses. Future studies could benefit from examining the same associations within specific labor activities. Although our sample size was large enough to ensure adequate statistical power, a larger sample could enhance the external validity for each work activity in subsequent investigations. Secondly, due to the cross-sectional design of our study, we were unable to explore changes over time or establish causal relationships. Therefore, future research should consider longitudinal designs to address these limitations. Additionally, the generalizability of our results is limited to Portuguese workers, and caution should be exercised when extrapolating the findings to other countries. As a self-report study, there is the possibility of bias in the data, particularly considering the potential influence of the pandemic on subjective experiences (e.g., office work vs. remote work). Lastly, we acknowledge a limitation regarding the lack of extensive sociodemographic data, such as socioeconomic status, level of education, marital status, type of contract, and shift work. While these factors could potentially influence the outcomes under investigation, they were not the primary focus of our study. Future investigations exploring the influence of sociodemographic variables on the variables of interest would benefit from incorporating these measures to provide a more comprehensive understanding of the phenomenon.

## 5. Conclusions

The present study holds significant implications for health practitioners involved in public health and the well-being of workers. Firstly, our findings highlight the importance of adaptive coping strategies, specifically instrumental support, reinterpretation, and humor, in contributing to a higher level of life satisfaction. The mean scores for adaptive coping, problem-focused coping, and life satisfaction were consistently high, indicating that most workers naturally employ adaptive coping strategies in their daily lives, with the workplace playing a crucial role in their overall well-being. Health practitioners should pay close attention to the detrimental impact of mental disorders on the workplace and individuals’ well-being and assess and address these issues effectively and in a timely manner, providing support and promoting the adoption of coping mechanisms that enhance goal-oriented work and life satisfaction.

While this study provides valuable insights into the practical relevance of the findings within the context of mental health, it is important to note that it is not an intervention study. Therefore, we cannot establish causality regarding the impact of specific coping responses on various work activities and individuals. However, the results do demonstrate that reinterpretation, instrumental support, and the management of grief significantly contribute to promoting life satisfaction. Seeking support, planning, and cultivating strategies for cognitive well-being and accepting the current situation facilitate a positive appraisal of life and better coping with the demands of stressful events.

The outcomes of this study serve as a foundation for further exploration of the relationships between different coping strategies, symptoms of depression, anxiety, stress, and life satisfaction among individuals who experienced lockdowns while continuing to work. Those who confronted heightened stress and anxiety in challenging environments, may be inclined to employ more adaptive coping and problem-solving mechanisms, leading to greater life satisfaction. This study provides additional evidence supporting the pivotal role of adaptive coping factors in enhancing life satisfaction among Portuguese workers. Workers demonstrate persistence when depressive symptoms, anxiety, and stress are minimized, and when they employ adaptive coping strategies that foster life satisfaction.

## Figures and Tables

**Table 1:** Descriptive statistics, correlations, and internal consistency coefficients.

Variables	M	SD	S	K	1	2	3	4	5	6	7	8	9	10	11	12	13	14	15	16	17	α
1. Active Cope	3.73	0.91	−0.77	0.48																		0.82
2. Planning	3.98	0.80	−0.87	0.85	0.73 **																	0.68
3. Instrumental Support	3.20	0.95	−0.15	−0.59	0.26 **	0.36 **																0.79
4. Emotional Support	3.10	1.07	0.03	−0.79	0.20 **	0.24 **	0.50 **															0.85
5. Religion	2.25	1.16	0.76	−0.42	0.11 *	0.13 **	0.24 **	0.27 **														0.83
6. Reinterpretation	3.46	0.94	−0.18	−0.47	0.50 **	0.47 **	0.26 **	0.27 **	0.17 **													0.81
7. Self-Blame	3.32	0.84	−0.17	−0.04	0.18 **	0.22 **	0.22 **	0.20 **	0.03	0.11 *												0.76
8. Acceptance	3.32	0.85	−0.09	−0.45	0.28 **	0.31 **	0.23 **	0.19 **	0.12 *	0.34 **	0.15 **											0.73
9. Venting	3.20	1.01	−0.06	−0.27	0.15 **	0.27 **	0.27 **	0.36 **	0.14 **	0.12 *	0.25 **	0.21 **										0.86
10. Denial	2.27	0.89	0.49	−0.08	−0.078	−0.03	0.15 **	0.15 **	0.18 **	−0.02	0.21 **	0.07	0.38 **									0.81
11. Self-Distraction	2.99	1.00	0.07	−0.62	0.047	0.12 *	0.15 **	0.26 **	0.08	0.19 **	0.20 **	0.23 **	0.26 **	0.24 **								0.79
12. Disengagement	1.69	0.79	1.19	1.22	−0.24 **	−0.23 **	−0.13 **	−0.01	0.01	−0.16 **	0.13 **	−0.02	0.02	0.25 **	0.22 **							0.86
13. Substance Use	1.30	0.59	1.72	1.64	−0.07	−0.08	0.03	0.08	0.00	−0.04	0.17 **	0.02	0.14 **	0.23 **	0.08	0.27 **						0.84
14. Humor	2.74	0.97	0.34	−0.31	0.14 **	0.14 **	0.02	0.04	−0.05	0.34 **	0.09	0.25 **	0.08	0.03	0.32 **	0.14 **	0.14 **					0.82
15. Depressive Symptoms	1.45	0.48	1.40	1.96	−0.15 **	−0.11 *	0.02	0.06	−0.01	−0.25 **	0.23 **	−0.12 *	−0.02	0.17 **	0.11 *	0.25 **	0.21 **	−0.05				0.75
16. Anxiety Symptoms	1.36	0.41	1.77	1.52	−0.16 **	−0.14 **	0.05	0.04	0.02	−0.18 **	0.18 **	−0.12 *	0.00	0.17 **	0.13 *	0.23 **	0.23 **	−0.04	0.75 **			0.70
17. Stress Symptoms	1.44	0.44	1.06	0.69	−0.10	−0.09	0.05	0.04	−0.05	−0.18 **	0.23 **	−0.07	0.04	0.23 **	0.10	0.20 **	0.20 **	−0.07	0.74 **	0.69 **		0.74
18. Satisfaction with Life	3.61	1.05	0.40	0.10	0.19 **	0.15 **	0.14 **	−0.01	0.05	0.26 **	−0.13 **	0.13 *	0.01	−0.06	−0.11 *	−0.28 **	−0.13 **	−0.07	−0.35 **	−0.20 **	−0.11 *	0.84

Notes: M = Mean; SD = Standard-Deviation; S = Skewness; K = Kurtosis; below diagonal line = bivariate correlations; above diagonal line = partial correlations controlling for sex; α = Internal consistency coefficient; * *p* < 0.01; ** *p* < 0.01.

**Table 2:** Standardized beta coefficients and explained variance.

Model	β	t	*R* ^2^
Model 1			0.17
Depressive Symptoms	−0.61 **	−7.86	
Anxiety Symptoms	0.04	0.61	
Stress Symptoms	−0.31 **	4.38	
Model 2			0.27
Depressive Symptoms	−0.49 **	−6.39	
Anxiety Symptoms	0.06	0.85	
Stress Symptoms	−0.30 **	4.30	
Active Cope	0.04	0.64	
Planning	−0.02	−0.31	
Instrumental Support	0.11 *	2.07	
Emotional Support	−0.08	−1.53	
Religion	0.01	0.11	
Reinterpretation	0.20 **	3.46	
Self-Blame	−0.10	−1.96	
Acceptance	0.06	1.30	
Venting	−0.01	−0.13	
Denial	0.02	0.40	
Self-Distraction	−0.06	−1.12	
Disengagement	−0.13 *	−2.69	
Substance Use	−0.02	−0.44	
Humor	0.11 *	−2.19	

Notes: β = standardized coefficients; t = *t*-test; *R*^2^ = r-squared; * *p* < 0.01; ** *p* < 0.01.

## Data Availability

The data were used under license exclusively for the current study. The data that support the findings of this study are not publicly available but are available upon reasonable request and with permission of the Life Quality Research Center and the corresponding author.
